# Ending the evidence gap for pregnancy, HIV and co‐infections: ethics guidance from the PHASES project

**DOI:** 10.1002/jia2.25846

**Published:** 2021-12-15

**Authors:** Anne Drapkin Lyerly, Richard Beigi, Linda‐Gail Bekker, Benjamin H. Chi, Susan E. Cohn, Dázon Dixon Diallo, Joseph Eron, Ruth Faden, Elana Jaffe, Angela Kashuba, Mary Kasule, Carleigh Krubiner, Maggie Little, Joseph Mfustso‐Bengo, Lynne Mofenson, Victor Mwapasa, Lillian Mworeko, Landon Myer, Martina Penazzato, Annette Rid, Roger Shapiro, Jerome Amir Singh, Kristen Sullivan, Marissa Vicari, Jacque Wambui, Amina White, Marisha Wickremsinhe, Leslie Wolf

**Affiliations:** ^1^ Department of Social Medicine and Center for Bioethics University of North Carolina Chapel Hill North Carolina USA; ^2^ Department of Obstetrics Gynecology & Reproductive Sciences UPMC Magee‐Women's Hospital Pittsburgh Pennsylvania USA; ^3^ Desmond Tutu HIV Centre and Department of Medicine University of Cape Town Cape Town South Africa; ^4^ Department of Obstetrics and Gynecology University of North Carolina Chapel Hill North Carolina USA; ^5^ Department of Medicine Feinberg School of Medicine Northwestern University Chicago Illinois USA; ^6^ SisterLove, Inc Atlanta Georgia USA; ^7^ SisterLove, Inc Johannesburg South Africa; ^8^ Department of Medicine and Center for AIDS Research University of North Carolina Chapel Hill North Carolina USA; ^9^ Johns Hopkins Berman Institute of Bioethics Baltimore Maryland USA; ^10^ Eshelman School of Pharmacy and Department of Medicine University of North Carolina Chapel Hill North Carolina USA; ^11^ Botswana‐Baylor Centre for Clinical Excellence Gabarone Botswana; ^12^ Center for Global Development Washington DC USA; ^13^ Kennedy Institute for Ethics and Department of Philosophy Georgetown University Washington DC USA; ^14^ Center of Bioethics for Eastern & Southern Africa and Department of Health Systems and Policy College of Medicine University of Malawi Zomba Malawi; ^15^ Elizabeth Glaser Pediatric AIDS Foundation Washington DC USA; ^16^ College of Medicine University of Malawi Zomba Malawi; ^17^ International Community of Women Living with HIV Eastern Africa Kampala Uganda; ^18^ Division of Epidemiology and Biostatistics School of Public Health and Family Medicine University of Cape Town Cape Town South Africa; ^19^ HIV Department World Health Organization Geneva Switzerland; ^20^ Department of Bioethics The Clinical Center National Institutes of Health Bethesda Maryland USA; ^21^ Department of Immunology and Infectious Diseases Harvard T.H. Chan School of Public Health Boston Massachusetts USA; ^22^ Howard College School of Law University of KwaZulu‐Natal KwaZulu‐Natal South Africa; ^23^ Dalla Lana School of Public Health Sciences, University of Toronto Toronto Ontario Canada; ^24^ International AIDS Society Geneva Switzerland; ^25^ National Empowerment Network of People Living with HIV and AIDS in Kenya (NEPHAK) African Communities Advisory Board (AfroCAB) Lusaka Zambia; ^26^ Department of Obstetrics and Gynecology University of North Carolina Chapel Hill North Carolina USA; ^27^ Ethox Centre and Wellcome Centre for Ethics and Humanities University of Oxford Oxford UK; ^28^ Center for Law Health & Society and College of Law and School of Public Health Georgia State University Atlanta Georgia USA

**Keywords:** co‐infections, ethics, HIV, pregnancy, prevention, research

## Abstract

**Introduction:**

While pregnant people have been an important focus for HIV research, critical evidence gaps remain regarding prevention, co‐infection, and safety and efficacy of new antiretroviral therapies in pregnancy. Such gaps can result in harm: without safety data, drugs used may carry unacceptable risks to the foetus or pregnant person; without pregnancy‐specific dosing data, pregnant people face risks of both toxicity and undertreatment; and delays in gathering evidence can limit access to beneficial next‐generation drugs. Despite recognition of the need, numerous barriers and ethical complexities have limited progress. We describe the process, ethical foundations, recommendations and applications of guidance for advancing responsible inclusion of pregnant people in HIV/co‐infections research.

**Discussion:**

The 26‐member international and interdisciplinary Pregnancy and HIV/AIDS: Seeking Equitable Study (PHASES) Working Group was convened to develop ethics‐centred guidance for advancing timely, responsible HIV/co‐infections research with pregnant people. Deliberations over 3 years drew on extensive qualitative research, stakeholder engagement, expert consultation and a series of workshops. The guidance, initially issued in July 2020, highlights conceptual shifts needed in framing research with pregnant people, and articulates three ethical foundations to ground recommendations: equitable protection from drug‐related risks, timely access to biomedical advances and equitable respect for pregnant people's health interests. The guidance advances 12 specific recommendations, actionable within the current regulatory environment, addressing multiple stakeholders across drug development and post‐approval research, and organized around four themes: building capacity, supporting inclusion, achieving priority research and ensuring respect. The recommendations describe strategies towards ethically redressing the evidence gap for pregnant people around HIV and co‐infections. The guidance has informed key efforts of leading organizations working to advance needed research, and identifies further opportunities for impact by a range of stakeholder groups.

**Conclusions:**

There are clear pathways towards ethical inclusion of pregnant people in the biomedical research agenda, and strong agreement across the HIV research community about the need for – and the promise of – advancing them. Those who fund, conduct, oversee and advocate for research can use the PHASES guidance to facilitate more, better and earlier evidence to optimize the health and wellbeing of pregnant people and their children.

## 1 INTRODUCTION

Since the early 1990s, the management of pregnancy has been an important focus for HIV research. The urgent need to identify interventions to prevent perinatal transmission led to remarkable progress towards its elimination: with effective antiretroviral treatment and other interventions, the global rate of perinatal transmission can be reduced to 5% or lower [1].

Yet, critical evidence gaps remain. Pregnant people [2, 3] have been excluded from large trials of pre‐exposure prophylaxis [4, 5, 6, 7, 8] – even as pregnancy increases the risk of HIV infection up to three‐fold per sex act [9]. Pregnancy has been an exclusion criterion for trials of new antiretrovirals [10, 11] and drugs to treat malaria and tuberculosis [12, 13, 14] – even as HIV is associated with 6–20% of maternal deaths worldwide, and is especially deadly where co‐infection occurs [15]. Extensive post‐approval delays and a tendency to focus on foetal outcomes without due regard for maternal health limit pregnancy‐specific data. Pregnant people are among those most in need of drugs for safe and effective prevention and treatments of HIV and co‐infections, yet among those least likely to have timely, robust evidence to inform their care.

Harms of these evidence gaps are now widely recognized. Without timely pregnancy‐specific data, drugs carrying unacceptable risk to the pregnant person or foetus may be used in clinical practice. Without pharmacokinetic data specific to pregnancy, pregnant people may be underdosed, exposing them and their offspring to inadequately treated disease, or overdosed, exposing them to drug‐related toxicities [16]. Moreover, limited data can lead to delays in pregnant people's access to next‐generation drugs offering improved effectiveness and tolerability [17].

Leading researchers and organizations now affirm the need for responsible research with pregnant people, in general and in the context of HIV [18, 19, 20, 21, 22, 23, 24, 25, 26, 27, 28]. Yet, evidence gaps reflect a long history of exclusionary practices, including a lack of incentives (e.g. financial) and requirements in drug approval pathways, problems in reasoning about research in pregnancy and patterns of thinking around pregnancy generally, such as the tendency to view pregnant people as “vessels and vectors” [29, 30, 31].

The Pregnancy and HIV/AIDS: Seeking Equitable Study (PHASES) Project was launched in 2013 to help shift the paradigm towards responsible inclusion of pregnant people in HIV/co‐infections research. A 26‐member interdisciplinary international Working Group was convened to develop the pinnacle product of this effort – ethics‐centred guidance, initially released in July 2020, entitled *Ending the Evidence Gap for Pregnant Women around HIV and Co‐infections: A Call to Action* [32]. The guidance has since informed key efforts by leading organizations working to advance needed research, including UNAIDS/WHO ethics guidance for HIV prevention emphasizing fair selection of subjects, inclusive of pregnancy [33]; an ongoing WHO and International Maternal Pediatric Adolescent AIDS Clinical Trials Network (IMPAACT) consensus development process for accelerating study of new drugs in pregnancy [26]; a Microbicide Trials Network (MTN) protocol for antiretroviral (ARV)‐based prevention in pregnancy [34]; and efforts addressing the need for pregnancy‐specific data by the U.S. Food and Drug Administration [35]. Here, we describe the process, ethical foundations and recommendations of the guidance, which provides a concrete, actionable pathway towards advancing timely, needed, responsible research with pregnant people. We highlight uptake of the guidance since its launch, and future opportunities for impact. While developed with specific attention to HIV/co‐infections, the guidance offers important lessons for other disease contexts, including the ongoing COVID‐19 pandemic.

## 2 DISCUSSION

The PHASES Working Group, convened to develop the guidance, reflected expertise in bioethics, public health, law, obstetrics and maternal‐foetal medicine, paediatrics, HIV research, infectious disease, pharmacology and community advocates for women living with HIV. Members were from Botswana, Kenya, Malawi, South Africa, Switzerland, Uganda, the United Kingdom and the United States. Deliberations occurred over approximately 3 years, including an in‐person meeting in 2018. Guidance was informed by qualitative research engaging pregnant people in the United States and Malawi [35, 36, 37]; commissioned country‐specific legal briefs; workshops with international representatives; consultations with over 150 subject area experts; and feedback on drafts from key stakeholders (e.g. community advisors, research oversight committee members, researchers, ethicists, clinicians and policy makers).

### 2.1 Conceptual shifts and ethical foundations

The guidance identifies a trio of *conceptual shifts* for the ethical framing of research.

The first shift is from viewing pregnant populations as “vulnerable” to viewing them as “complex.” The term vulnerable was otherwise applied to populations either judged unable to give valid consent or subject as a class to exploitation – neither of which applies to pregnancy, and had an unintentional chilling effect on research involving pregnant people [38]. Ethical and regulatory guidance documents have withdrawn “vulnerable” as a designation for pregnant people [18, 39], some endorsing the term “complex” to capture the physiologic differences and ethical complexities of research in pregnancy [18, 22].

Second is the shift from an emphasis on protecting pregnant people *from* research to protecting them *through* research. Protection from research risks is important, but failing to conduct research can also increase risk: without data collected in research settings, potential risks of drugs are exported to the clinical context, where they affect countless individuals. Moreover, exclusion from research may prevent pregnant people from accessing potentially beneficial healthcare interventions. As a recent example, access to rapid advances in SARS‐CoV vaccines and therapeutics has been relatively limited for pregnant people, as data on efficacy, safety and dosing data are lacking due to their exclusion from trials [40, 41]. Ultimately, advancing the health of pregnant people and their offspring requires responsibly conducted research that generates evidence for improving their care.

Third is the shift from presumptive exclusion to fair inclusion. Justice in research requires not only fair distribution of research burdens, but also fair distribution of research benefits [42]. Pregnant people as a population, and their interests, deserve equitable inclusion in the research agenda. This requires fair representation in public and private investments in efforts to generate evidence informing safe and effective use of a drug, especially given urgent health needs.

Building on these shifts, the ethical responsibility to address inequities in the evidence base for the use of medications in pregnancy is grounded on three *ethical foundations*: equitable protection, access and respect.

One, pregnant people deserve *equitable protection* from drug‐related risks [43]. A key mission of research is to gather evidence under controlled circumstances to mitigate risks when drugs are used in clinical care. Pregnant people are equally deserving of such protection for themselves and their offspring. While delays in gathering data for sub‐populations are common, delays for pregnancy are extensive, disproportionate to need and without adequate processes to mitigate them [16]. Research is essential to realizing the fundamental public health obligation to ensure approved drugs meet acceptable safety thresholds for those who will use them.

Two, pregnant people deserve timely *access* to medicine's most effective advances. Yet, lack of data creates obstacles to access. Limited or absent human data may lead to reticence among providers, practice guidelines and health systems to endorse the use of new drugs in pregnancy. These decisions often fail to consider advantages of next‐generation drugs over older formulations [17]. Incomplete data signalling possible but unproven risk can harden into restrictive policies that may endure for years.

Three, pregnant people deserve equitable *respect* for their own health. When research is conducted, it is critical that attention to foetal and child outcomes does not overshadow attention to maternal outcomes. While drugs are prescribed in pregnancy in part to benefit the developing child, decisions about use should also reflect due consideration of maternal health. Adequate data about maternal health outcomes are needed to inform the calculus.

Organizations have begun to apply these ethical considerations in promoting research to generate pregnancy‐specific evidence [44, 45] and identifying optimal drug regimens [3], modelling how ethics can serve to justify research priorities and shift presumptions going forward. Researchers and pharmaceutical companies working to advance innovative trials involving pregnant people [46, 47, 48, 49] can also use these considerations to guide future studies.

### 2.2 Recommendations

**Figure 1:**
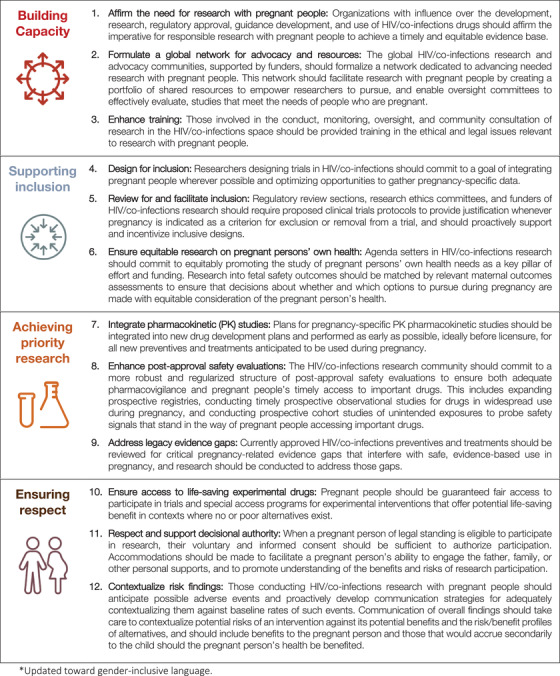
Recommendations of the PHASES guidance

The PHASES guidance further outlines 12 specific recommendations, derived from the ethical foundations of equitable protection, access and respect, and actionable within the current regulatory context. They address multiple stakeholders across the arc of drug development and post‐approval research, and are organized around four themes: building capacity, supporting inclusion, achieving priority research and ensuring respect (Figure 1).

The first three recommendations address the need to *build capacity* within the HIV research community. Currently, patterns of exclusion go beyond what might be expected from existing regulatory and ethical constraints, suggesting barriers within the research culture [24, 50]. Stakeholders and agenda‐setters can facilitate a cultural shift by affirming the need to conduct research with pregnant persons [Recommendation 1] – for instance by issuing public statements [28, 51] or endorsing frameworks for accelerated inclusion [52, 53]. The guidance also recommends expansion of key resources: formalizing a global network for advocacy and resources to capitalize on existing advocacy efforts, tools and educational resources [Recommendation 2]; and enhancing training to mitigate misunderstandings about permissible conditions for research with pregnant people across organizations that fund, conduct or provide guidance or oversight for research [Recommendation 3], drawing on a range of excellent educational tools [54].

The next three recommendations address the need to *support inclusion* of pregnant people and their interests in research through design and oversight. As pregnant people are among those most in need of safe and effective interventions, trials should be designed to integrate pregnant participants and gather pregnancy‐specific data whenever possible [Recommendation 4]. Approaches include pursuing a variety of inclusive trial designs that either target pregnant people for enrolment or remove pregnancy from exclusion criteria; conducting preclinical animal reproductive toxicology studies earlier in drug development (e.g. phase 2); retaining individuals who become pregnant during clinical trials on study drug; capturing and analysing foetal and maternal outcomes for pregnancies occurring during trials; and further harmonizing data on maternal and foetal outcomes.

Oversight bodies and funders can likewise review for and facilitate the inclusion of pregnant people in research [Recommendation 5]. Strategies include issuing requirements for clear, specific justification whenever pregnancy is a proposed exclusion or removal criterion, and incentivizing inclusive research designs by encouraging inclusion in calls for funding and awards. Funders and agenda setters should commit to equity of attention and funding addressing pregnant people's own health needs, not just those of the foetus [Recommendation 6]: trials including pregnant people should identify opportunities to expand the scope of data collected to address maternal health and longer‐term outcomes for childbearing people; data and safety monitoring boards should ensure that stopping rules include relevant maternal and obstetric outcomes, and avoid disproportionately weighing neonatal over maternal outcomes; and guidelines recommending drug choice during pregnancy should consider implications for both maternal and infant health.

The next three recommendations are aimed at *achieving priority research*. Pregnancy‐specific pharmacokinetic studies should be integrated into new drug development plans and performed as early as possible, ideally before licensure, for new drugs anticipated to be used in pregnancy [Recommendation 7]. Another priority is safety. Absent in‐human data, concerns about the safety of drugs for the foetus often obstruct pregnant people's timely access to needed new drugs – potentially problematic for both maternal and foetal health. Since intervention studies may not accurately or adequately characterize the likelihood of rare adverse events, such as birth defects, the guidance recommends enhancing post‐approval safety evaluations [Recommendation 8] to address pregnancy‐specific limitations of most drug safety registries, which may lead to false alarms and restricted access to important drugs. Specific approaches include expanding prospective adverse event data collection through robust prospective modalities, such as the Antiretroviral Pregnancy Registry [55] and the WHO Registry for drug safety surveillance in pregnancy [56]; conducting post‐approval safety studies for drugs with widespread use in pregnancy; and committing to the timely pursuit of safety signals [57].

In addition to accelerating the evidence for new drugs, there is a need to address key legacy evidence gaps [Recommendation 9]. Absence of pregnancy‐specific evidence for currently available therapies may significantly affect access, equity or risk in the context of pregnancy. Public research institutes and private and industry funders can make a critical difference in directing funding to areas of greatest need, such as prevention [34]. Priority should be given to the most pressing or impactful gaps – those regarding drugs widely used in pregnancy but lacking evidence on maternal outcomes, or drugs widely used in non‐pregnant populations but lacking adequate safety data for woman and foetus; and pregnancy‐specific pharmacokinetic data on commonly used or urgently needed drugs.

The final recommendations centre on *ensuring respect* for pregnant people – for their immediate health needs, autonomy and interests in the interpretation and communication of research findings. One of the clearest cases is where participation in a trial or special access program offers the only prospect for life‐saving benefits. Previously, pregnancy has been used as an exclusion criterion for access to such trials or programs, even where no or poor alternatives exist and no risk to the foetus from the intervention has been identified [58, 59]. HIV/co‐infections researchers should ensure that pregnant people have fair access to potentially life‐saving experimental drugs [Recommendation 10] by removing pregnancy as exclusion criterion for access, unless there is demonstrable evidence that risks of the intervention outweigh potential benefits for pregnant people and their offspring.

In addition to respecting a pregnant person's health interests, it is critical to respect and support their decisional authority [Recommendation 11]. While many individuals (the father or partner, family members and personal supports) may have an interest in the outcome of pregnancy, a pregnant person of legal age should be at the centre of decisions and their voluntary and informed consent should be sufficient to authorize research participation. Researchers should provide meaningful decisional support to prospective participants, which may include facilitating consultations with partners and family, and work to mitigate social risks of participation, such as partner or family violence and abandonment.

The final element of respect regards responsible communication of research findings. Clear risk assessment, communication and translation are important for any research, but research in pregnancy brings special challenges and potential distortions. Adverse events, common in pregnancy, may be particularly alarming in the context of research. Such events should be anticipated and researchers should proactively develop communication plans that contextualize risk findings [Recommendation 12] against baseline rates in pregnancy and against the risks, benefits and uncertainties of alternatives. As research in pregnancy increases, contextualizing risk in publications and research communications will be critical to ensuring that studies lead to better health for pregnant persons and their children.

These 12 recommendations, grounded in ethics, are a resource for stakeholders working to improve care for pregnant people and their offspring through better evidence, and have informed calls for a more inclusive agenda [27, 60, 61]. Going forward, pharmaceutical companies can use the guidance to inform approaches to study design and research priorities; regulatory agencies can build on the recommendations in developing strategies for improving knowledge about pharmaceuticals in pregnancy; funders and agenda‐setters can cite guidance as they consider investment in and prioritization of research with pregnant people; oversight bodies can use the guidance in formulating a more ethical and inclusive approach to research protections; researchers can highlight recommendations that support important studies. Finally, the guidance can help scaffold burgeoning global advocacy by and for pregnant people to be included in responsible biomedical research.

## 3 CONCLUSIONS

The HIV community has a long history of finding pathways to address and improve the health of complex and underserved communities. The same creative and inclusive approach should be applied towards closing critical evidence gaps for pregnant populations. There are clear pathways forward and growing agreement about the need for – and promise of – advancing them. Those who fund, conduct, oversee and advocate for research can build on PHASES guidance and momentum to facilitate more, better and earlier evidence to optimize the health and wellbeing of pregnant people and their children.

## COMPETING INTERESTS

The authors declare no competing interests.

## AUTHOR'S CONTRIBUTIONS

ADL drafted the manuscript. ADL, RB, LGB, BHC, SEC, DDD, JE, RF, EJ, AK, MK, CK, ML, JMB, LM, VM, LM, LM, MP, AR, RS, JAS, KS, MV, JW, AW, MW and LW contributed to its development and provided important intellectual content. ADL, RB, LGB, BHC, SEC, DDD, RF, EJ, AK, MK, CK, ML, JMB, LM, VM, LM, LM, MP, AR, RS, JAS, KS, MV, JW, AW, MW and LW critically revised the manuscript. ADL, RB, LGB, BHC, SEC, DDD, JE, RF, EJ, AK, MK, CK, ML, JMB, LM, VM, LM, LM, MP, AR, RS, JAS, KS, MV, JW, AW, MW and LW read and approved the final manuscript.

## FUNDING

This work was supported by the National Institute of Allergy and Infectious Diseases of the National Institutes of Health under award number R01AI108368 (Lyerly PI). The work was also supported in part by the Clinical Center Department of Bioethics, which is in the Intramural Program of the National Institutes of Health. The views expressed here are those of the authors and do not necessarily reflect the policies of the National Institutes of Health or the U.S. Department of Health and Human Services.

## Data Availability

Data sharing is not applicable to this article as no new data were created or analyzed in this study.
